# Identification of discriminative neuroimaging markers for patients on hemodialysis with insomnia: a fractional amplitude of low frequency fluctuation-based machine learning analysis

**DOI:** 10.1186/s12888-022-04490-1

**Published:** 2023-01-04

**Authors:** Ze-ying Wen, Yue Zhang, Meng-han Feng, Yu-chi Wu, Cheng-wei Fu, Kan Deng, Qi-zhan Lin, Bo Liu

**Affiliations:** 1 The Second Clinical College, Guangzhou University of Chinese Medicine, Guangzhou, 510120 China; 2 Department of Radiology, The First Affiliated Hospital of Henan University of Chinese Medicine, Zhengzhou, 450000 China; 3 Department of Radiology, The Second Affiliated Hospital of Guangzhou University of Chinese Medicine, Guangzhou, 510120 China; 4 R&D Support Group, Xin-Huangpu Joint Innovation Institute of Chinese Medicine in Guangdong Province, Guangzhou, 510700 China; 5 Hemodialysis Department, The Second Affiliated Hospital of Guangzhou University of Chinese Medicine, Guangzhou, 510120 China; 6 Philips Healthcare, Guangzhou, 510120 China

**Keywords:** Hemodialysis, Insomnia, Amplitude of low frequency fluctuation, Support vector machine (SVM), Neural markers

## Abstract

**Background and objective:**

Insomnia is one of the common problems encountered in the hemodialysis (HD) population, but the mechanisms remain unclear. we aimed to (1) detect the spontaneous brain activity pattern in HD patients with insomnia (HDWI) by using fractional fractional amplitude of low frequency fluctuation (fALFF) method and (2) further identify brain regions showing altered fALFF as neural markers to discriminate HDWI patients from those on hemodialysis but without insomnia (HDWoI) and healthy controls (HCs).

**Method:**

We compared fALFF differences among HDWI subjects (28), HDWoI subjects (28) and HCs (28), and extracted altered fALFF features for the subsequent discriminative analysis. Then, we constructed a support vector machine (SVM) classifier to identify distinct neuroimaging markers for HDWI.

**Results:**

Compared with HCs, both HDWI and HDWoI patients exhibited significantly decreased fALFF in the bilateral calcarine (CAL), right middle occipital gyrus (MOG), left precentral gyrus (PreCG), bilateral postcentral gyrus (PoCG) and bilateral temporal middle gyrus (TMG), whereas increased fALFF in the bilateral cerebellum and right insula. Conversely, increased fALFF in the bilateral CAL/right MOG and decreased fALFF in the right cerebellum was observed in HDWI patients when compared with HDWoI patients. Moreover, the SVM classification achieved a good performance [accuracy = 82.14%, area under the curve (AUC) = 0.8202], and the consensus brain regions with the highest contributions to classification were located in the right MOG and right cerebellum.

**Conclusion:**

Our result highlights that HDWI patients had abnormal neural activities in the right MOG and right cerebellum, which might be potential neural markers for distinguishing HDWI patients from non-insomniacs, providing further support for the pathological mechanism of HDWI.

**Supplementary Information:**

The online version contains supplementary material available at 10.1186/s12888-022-04490-1.

## Introduction

Patients receiving maintenance hemodialysis (HD) frequently report insomnia complaints, with a high prevalence ranging from 40 to 85% worldwide [1–3]. It has been shown that HD patients with insomnia (HDWI) present a variety of comorbidities such as irritability, immune suppression, anxiety, depression, cognitive impairment, etc., which may have a potentially great impact on their quality of life and even survival [4–6]. Despite the fact that the current management including medication, behavioral cognitive therapy (CBT-i) and acupuncture has developed for HDWI, it is far from satisfactory and standardized clinical procedures regardless of individual differences may increase the subjects’ risk factors amongst HD patients [7–10], highlighting the urgent need to fully understand the pathophysiology of the disorder and help achieve advances in the prevention and treatment of the condition.

Resting-state functional magnetic resonance imaging (rs-fMRI) is a non-invasive technique, which could detect the ongoing neuronal process at the “resting state” through measuring the spontaneous brain activity by low-frequency fluctuations in blood oxygen level-dependent (BOLD) signals, and consequently provide a new opportunity to investigate the functional abnormalities on several neurological disorders [11–13]. In recent years, rs-fMRI studies have identified that the altered amplitude of low-frequency fluctuations (ALFF) or fractional fractional amplitude of low frequency fluctuation (fALFF), which reflect the intensity of spontaneous neuronal activity in local brain regions, underlies insomnia [14]. Interestingly, accumulative rs-fMRI evidence has suggested that patients undergoing HD are associated with aberrant neuronal activities in widespread brain regions, including the sensorimotor network (SMN) regions, default mode network (DMN) regions, temporal lobe, amygdala, etc. [15–17]. However, despite a large body of empirical research in HD subject groups as has demonstrated, we noticed that results emphasizing on the specific research colony of HDWI cohort are still lacking. Furthermore, these previous studies mainly focused on the group-level investigations, calling for more studies to be performed to pinpoint the distinct brain features for HDWI, that could be translated into reliable individual-level diagnostic biomarkers and help us better understand the pathophysiological mechanisms of diseases.

In the past several years, there has been a promising improvements of machine learning (ML) techniques in brain disease classification or prediction. The strength of ML algorithm is that it could detect hard-to-discern patterns from the large and complex data sets and is particularly well-suited to large fMRI data mining, especially in exploring neurological disease biomarkers for disease diagnosis and underlying mechanisms [18–20]. There are two types of ML algorithm at present—supervised and unsupervised learning. As one of supervised ML techniques that builds a model by learning from known classes, As one of ML techniques, the support vector machine (SVM) has the potential to capture the voxel covariance patterns of BOLD responses to fMRI with a least absolute shrinkage and selection operator (LASSO) and to construct a cross-validated model for group classification [21, 22]. It has been demonstrated that when applying SVM classifier to characterize neurological disorders using diverse features from rs-fMRI data, an excellent performance could be obtained [23–25]. Moreover, rs-fMRI studies have identified that altered amplitude of low-frequency fluctuations (ALFF) or fractional fractional amplitude of low frequency fluctuation (fALFF), which reflect the intensity of spontaneous neuronal activity in local brain regions, underlies insomnia [24, 25]. Investigating the ALFF may advance our understanding of the spontaneous neural activities of the brain in HDWI patients at group-level, while the combination with ML algorithm may provide multi-level information for disease classification, and may give better understanding of the mechanisms of HDWI than single-level study.

Therefore, in this study, we attempted to employ SVM method to explore the discriminative neuroimaging biomarkers for HDWI. Specifically, we first compared the fALFF differences among HDWI subjects, HDWoI subjects and HCs, and investigated the correlation of the altered fALFF value with clinical measures. Then, based on the extracted fALFF features across groups, we constructed a SVM classifier to identify the most promising brain regions that could distinguish the HDWI patients from those non-insomniacs.

## Materials and methods

### Subjects

28 HD subjects comorbid with insomnia (HDWI), 28 HD subjects without insomnia (HDWoI), and 28 sex-, age-, education-matched healthy controls (HCs) were recruited between April 2021 and July 2022 from the Department of hemodialysis center, The Second Affiliated Hospital of Guangzhou University of Chinese Medicine (Guangdong Provincial Hospital of Chinese Medicine). This study was approved by the Institutional Review Board of the Second Affiliation Hospital, Guangzhou University of Chinese Medicine. Informed consent was also obtained from all participants.

### Inclusion &exclusion criteria

HDWI subjects meeting the following criteria were included: (1) aged between 18–80 years old; (2) patients receiving regular hemodialysis (two or three sessions every week, 4 h each session, total weekly dialysis period ≥ 10 h) and more than 3 months; (3) insomnia diagnosed according to The Diagnostic and Statistical Manual of Mental Disorders, Fifth Edition(DSM-5) [26]; (4) baseline global Pittsburgh Sleep Quality Index (PSQI) score ≥ 7, Self-Rating Anxiety Scale (SAS) score ≤ 59, and Self-Rating Depression Scale (SDS) score ≤ 59. (5) voluntary participants and informed consent signed. The exclusion criteria included: (1) patients with a history of cancer, congestive heart failure, connective tissue disease and hematological diseases and other serious comorbidities; (2) inadequately dialyzed, indicated by urea clearance index (KT/V) < 1.20; (3) contraindications to MRI scanning and inability to complete the neuropsychological test; and (4) translational motion greater than 2.5 mm, rotation greater than2.5^◦^.

### Neuropsychological assessment

Neuropsychological assessments were performed in all subjects before the MR scan on the day before HD treatment. The Pittsburgh sleep quality index(PSQI) was employed to evaluate the subjects’ sleep function [27]. It includes seven items: sleep quality, sleep latency, sleep duration, sleep efficiency, sleep disturbance, hypnotic use and daytime dysfunction. It should be noted that the patients recruited in this study were asked to stop using hypnotic medication during the whole study, thus the sub-component of hypnotic use was not regarded as outcome measurement. According to the Diagnostic and Statistical Manual of Mental Disorders, Fifth Edition(DSM-5), insomnia were defined as PSQI score ≥ 7. The 20-item Self-Rating Anxiety Scale (SAS) and 20-item self-rating depression scale (SDS) were used to assess the subjects’ anxiety and depression status, respectively [28]. In addition, the short form 36 health survey questionnaire (SF-36) was also be used as the secondary outcome measure to assess the patients’ quality of life from 8 sections including physical functioning, role physical, bodily pain, general health, vitality, social functioning, role emotional, mental health [29].

### fMRI data acquisition

fMRI scanning was performed on a 3.0-T Ingenia MR scanner (Philips, Amsterdam, Netherlands) with a 32-channel birdcage head coil. To minimize head movement and scanner noise, foam padding and earplugs were applied. All subjects were required to remain motionless, and keep their eyes closed but be awake. All of them participated in the identical functional MRI (fMRI) scanning sessions 24 h after the hemodialysis. The fMRI parameters were as follows: (1) T1- weighted structural images: repetition time (TR) = 1.0 ms, echo time (TE) = 4.7 ms, field of view (FOV) = 256 × 240 × 224 mm, matrix = 320 × 300 × 280 slices, voxel size = 0.8 × 0.8 × 0.8 mm, flip angle = 9^◦^,dynamic scans = 240, slices = 280, slice gap = 0 mm, slice thickness = 1.0 mm. (2) Resting-state fMRI images: TR = 2,000 ms, TE = 30 ms, FOV = 240 × 240 × 142 mm, matrix = 64 × 61 × 38 slices, voxel size = 3.75 × 3.75 × 3.5 mm, flip angle = 9^◦^, dynamic scans = 240, slices = 38, slice gap = 0.25 mm.

### fMRI preprocessing and fALFF analysis

The rs-fMRI data were preprocessed in Data Processing and Analysis for Brain Imaging 3.0 (DPABI 3.0) [30]. The details of scanning parameters preprocessing steps were Similar to our previous study [31]. In brief, the preprocessing procedures included the following steps: removal of the first 10 time points; slice timing and realignment (subjects with head motion > 2.5 mm or > 2.5^◦^were excluded); standardization of the functional and structural images into Montreal Neurological Institute (MNI) space; spatial normalization and resampling(3 × 3 × 3 mm^3^); smooth with a 6-mm full-width-half-maximum Gaussian kernel; temporally filtering (0.01–0.08 Hz) to generate the ALFF value, and then, the fALFF map was obtained by dividing the total ALFF values from 0.01 to 0.025 Hz; and transforming the fALFF map to the z-fALFF map with normal z transformation.

As for the fALFF statistical analyses, we employed a one-way analysis of covariance ( ANOVA) to calculate the difference of fALFF (z value) among three groups, with age, sex, and head motion as covariates. A threshold of voxel-wise *p* < 0.001 uncorrected and cluster-level *p* < 0.05 after 3dFWHMx and 3dClustSim [AFNI (https://afni.nimh.nih.gov/) released in July 2017] was applied for the fALFF analyses. Th*e post-hoc* tests were further used for pairwise comparison. Also, the person’s correlation coefficient was used to explore the relationship between the changed fALFF value and neuropsychological assessments.

### Machine learning analyses

Based on the selected features that showed as regions of interest (ROIs), we constructed a SVM classifier (SVM, provided by the LIBSVM toolkit) [32]. In this study, we took all meaningful voxels within ROIs with the highest ranks to calculate the accuracy, setting the step until incorporating all features. The.

**Fig. 1:**
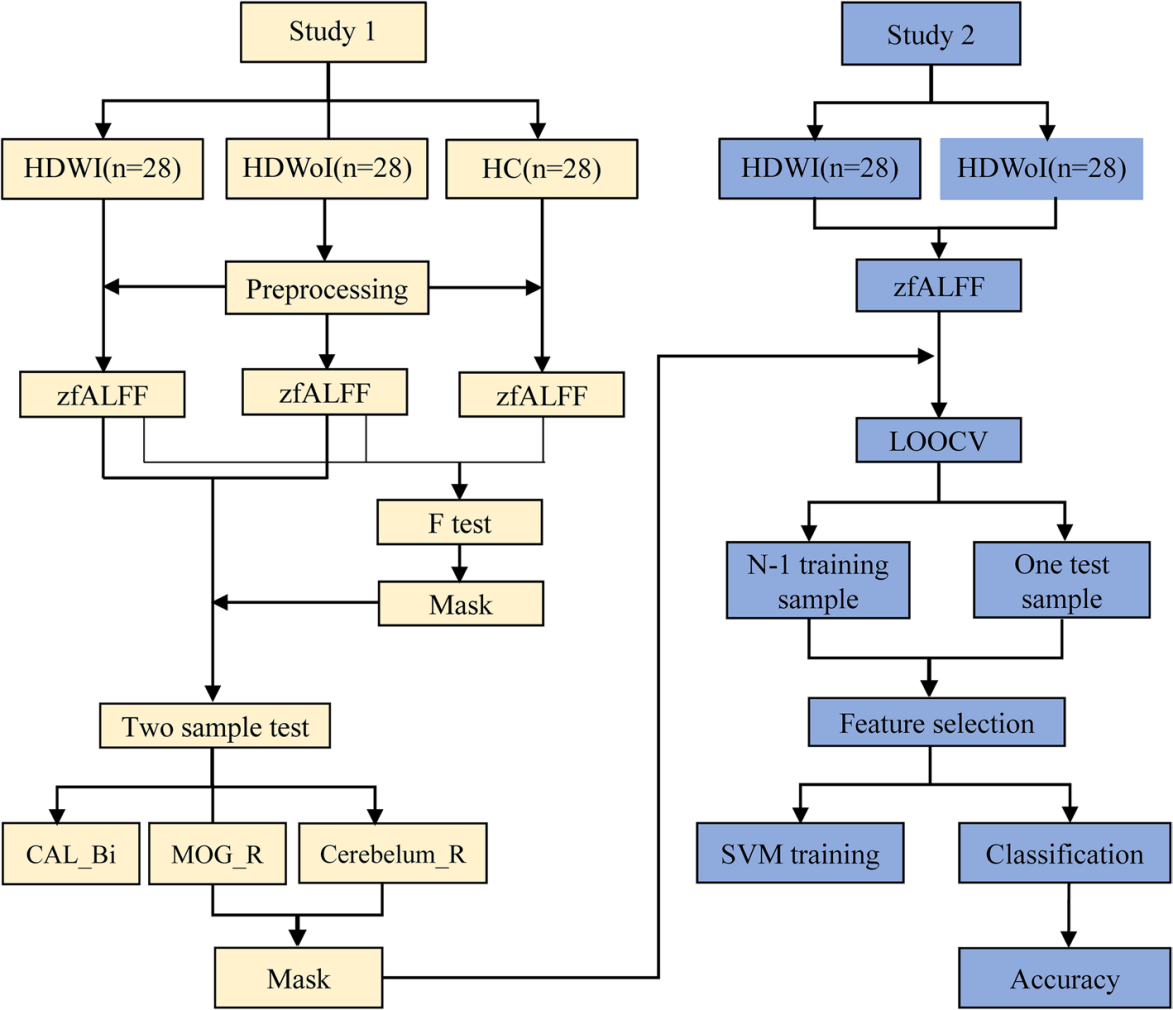
The flow diagram of classification. Study 1: fMRI preprocessing and fALFF analysis; Study 2: Machine learning analyses. HDWI = hemodialysis with insomnia; HDWoI = hemodialysis without insomnia; HC = healthy controls; fALFF = fractional fractional amplitude of low frequency fluctuation; CAL = calcarine; MOG = middle occipital gyrus; Bi = bilateral; R = right; LOOCV = leave-one-out cross-validation; SVM = support vector machine

First, based on the group-level ANOVA on fALFF values among three groups, significant differences for fALFF were retained as input features for the subsequent analyses to construct a SVM classifier (SVM, provided by the LIBSVM toolkit) [32]. Second, the leave-one-out cross-validation (LOOCV) method was used to reduce the risk of over-fitting, and the performance of classifier was quantified by accuracy, area under the curve (AUC), sensitivity, and specificity. To assess the robustness of the model, a non-parametric permutation test (permutation times = 5000) was performed as well, and the significance threshold was set to *p* < 0.05 (two-tailed). Finally, after obtaining the best-performing model, we extracted all discriminative features of the model, and thus identified a spatial representation of the regions that contributed most to the group discrimination as robust neural markers. See Fig. 1 for the flow diagram of classification.

### Statistical analysis for clinical variables

Clinical variables including gender, age, education, body mass index (BMI), hemodialysis duration and neuropsychological assessments were collected and analyzed using SPSS 22.0 software package (IBM Corp., Armonk, NY, USA). Continuous variables were assessed for normality by the Shapiro–Wilk test and visualization by histogram (not reported). If normally distributed, these continuous variables were calculated with a one-way analysis of variance (ANOVA) test and reported as mean ± standard deviation (mean ± SD). Otherwise, Kruskal–Wallis test was used and the median plus interquartile range were reported. Categorical measures were assessed using Chi-square test. The significance level of this study was set as 0.05, two-tailed, and post hoc tests (*p* < 0.05, Bonferroni corrected) were further used for pairwise comparison.

## Results

### Demographic and clinical measurements

The demographic and clinical characteristics of all the subjects are summarized in Table 1. There were no significant differences in age, sex, and education among three groups( *p* > 0.05). The PSQI total score, PSQI subscore, SDS score, SAS score and SF-36 score in HDWI subjects were significantly higher than HDWoI subjects and HCs, respectively (*p* < 0.001,). Furthermore, there were no significant difference in hemodialysis duration between HDWI group and HDWoI group.

**Table 1:** Subjects’ demographic and clinical characteristics

Variables	HDWI	HDWoI	HCs		*Post-hoc*		
				*p*	*p1*	*p2*	*p3*
Gender(male)^&^	13	15	13	0.827	-	-	-
Age(year)^#^	56.86 ± 11.10	55.11 ± 13.76	51.96 ± 14.45	0.376	-	-	-
Education^&^ (≤ Junior/Senior/ ≥ College)	9/15/14	12/9/7	10/7/11	0.248	-	-	-
BMI(kg/m^2^)*	20.56 (19.36, 22.59)	22.41 (19.88, 24.26)	21.54 (20.27, 22.78)	0.182	-	-	-
HD duration(year)*	7.00 (3.25, 12.75)	2.75 (1.00, 6.00)	-	0.000	0.000	0.000	0.138
PSQI(score)*	17.00 (15.25, 18.75)	3.00 (2.00, 4.75)	4.00 (2.00, 4.75)	0.000	0.000	0.000	1.000
Sleep quality(score)*	3.00 (2.00, 3.00)	0.00 (0.00, 0.00)	1.00 (0.00, 1.00)	0.000	0.000	0.000	0.098
Sleep latency(score)*	3.00 (3.00, 3.00)	0.00 (1.00, 1.00)	0.00 (1.00, 1.00)	0.000	0.000	0.000	1.000
Sleep duration(score)*	3.00 (2.25, 3.00)	0.00 (0.00, 1.00)	0.00 (0.00, 1.00)	0.000	0.000	0.000	1.000
Sleep efficiency(score)*	3.00 (3.00, 3.00)	0.00 (0.00, 0.00)	0.00 (0.00, 0.00)	0.000	0.000	0.000	1.000
Sleep disturbances(score)*	2.00 (2.00, 2.00)	1.00 (0.25, 1.00)	1.00 (1.00, 1.00)	0.000	0.000	0.000	1.000
Day time dysfunction(score)*	3.00 (2.00, 3.00)	1.00 (0.00, 1.00)	1.00 (0.00, 1.00)	0.000	0.000	0.000	1.000
SDS(score)*	54.38 (46.25, 58.44)	38.13 (31.25, 43.13)	30.63 (26.25, 35.00)	0.000	0.000	0.000	0.086
SAS(score)*	44.38 (37.81, 50.94)	32.50 (29.06, 36.25)	31.88 (28.75, 37.50)	0.000	0.000	0.000	1.000
SF-36(score)*	447.75 (381.88, 623.88)	709.00 (626.38, 752.25)	774.75 (696.25, 791.75)	0.000	0.000	0.000	0.137

*HCs* healthy controls, *HD* hemodialysis, *HDWI* hemodialysis with insomnia, *HDWI* hemodialysis without insomnia, *BMI* body Mass Index, *PSQI* Pittsburgh sleep quality index, *SDS* self-rating depression, *SAS* self-rating anxiety scale, *SF 36* the short form 36 health survey questionnaire

^#^ AVOVA; * Kruskal–Wallis test*;* ^&^ Pearson’s chi-square test

*p*1 = HDWI vs HCs; *p*2 = HDWI vs HDWoI; *p*3 = HDoWI vs HCs

Continuous values are expressed as mean ± standard deviation or median [interquartile range (IQR)]

### fALFF analysis

**Fig. 2:**
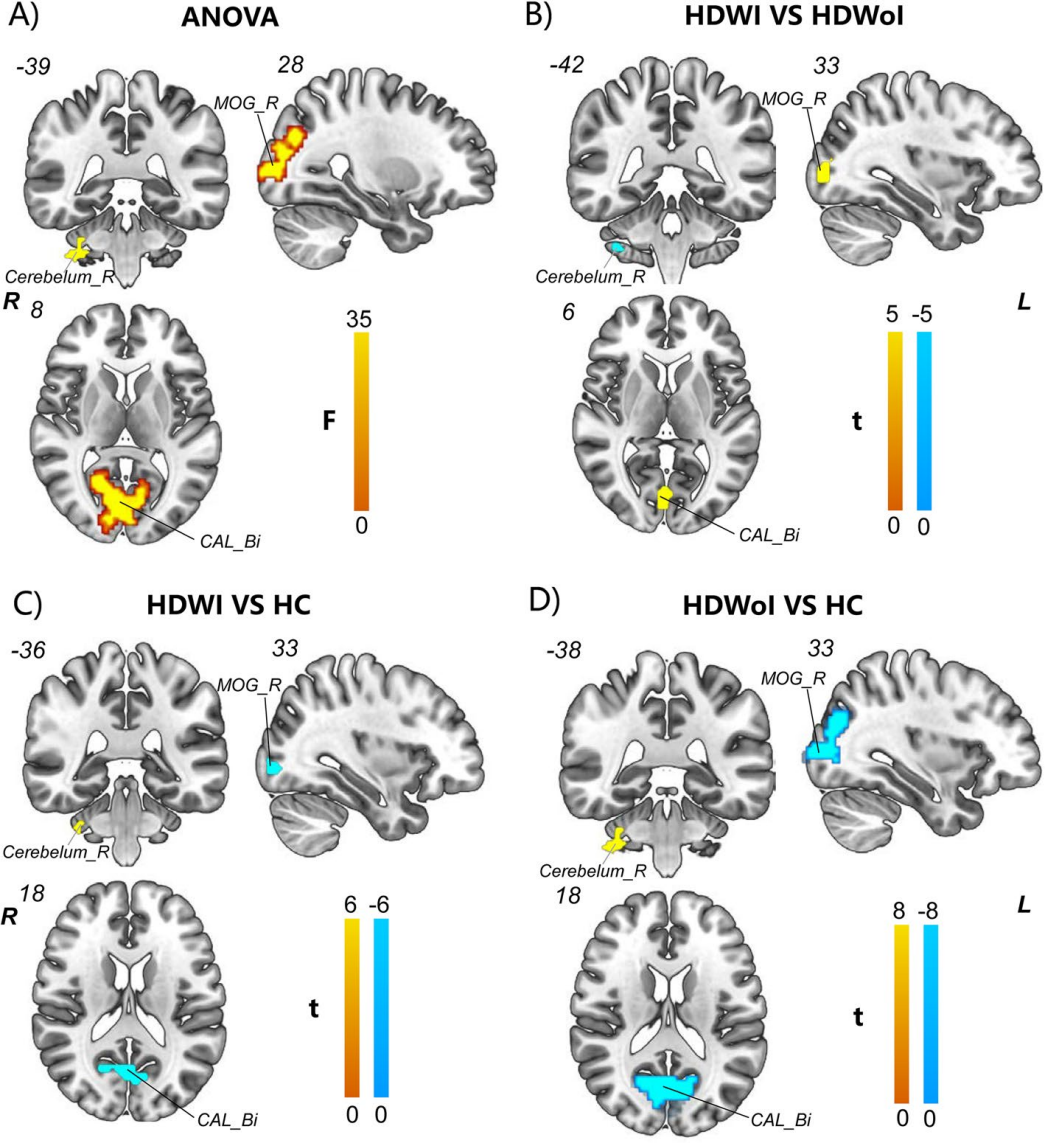
fALFF difference among and between groups **(A-D).** A) AVOVA analysis showed significant increased fALFF in the right MOG/ cerebellum and bilateral CAL among the three groups; B) Compared with HDWoI, HDWI subjects showed significant increased fALFF value in the right MOG and bilateral CAL whereas decreased fALFF value in the right cerebellum. C) Compared with HCs, HDWI subjects showed significant decreased fALFF value in the right MOG and bilateral CAL whereas increased fALFF value in the right cerebellum. D) Compared with HCs, HDoWI subjects showed significant decreased fALFF value in the right MOG and bilateral CAL whereas increased fALFF value in the right cerebellum. Abbreviation: Bi = bilateral; R = Right; CAL = calcarine; MOG = middle occipital gyrus; * = a threshold of voxel-wise *p* < 0.001 uncorrected and cluster-level *p* < 0.05; ANOVA: analysis of variance; yellow represents a significant increase and green represents a significant decrease

Analysis of ANOVA among the three groups revealed significant fALFF difference in multiple regions including the bilateral calcarine (CAL),bilateral middle occipital gyrus (MOG), bilateral precentral gyrus (PreCG), bilateral postcentral gyrus (PoCG), right lingual gyrus (LING), bilateral inferior inferior parietal lobule (IPL), left superior parietal (SPL), bilateral cerebellum, and right insula ( voxel-wise *p* < 0.001 uncorrected and cluster-level *p* < 0.05 after 3dFWHMx and 3dClustSim) (see Fig. 2 and Table 2).

**Table 2:** The fALFF differences among groups ( HDWI, HDWoI and HC, F test)

Region	MNI coordinates X Y Z			Peak z value	F value	Cluster size
CAL_Bi	3	-69	18	5.65	23.98	639
MOG_L	-33	-84	0	4.98	18.25	385
MOG_R	28	-87	8	6.56	33.90	228
PoCG/PreCG_L	-42	-15	59	5.17	19.78	243
PoCG/PreCG_R	63	-9	36	4.03	20.35	269
LING_R	12	-87	-3	6.56	30.12	285
TMG_L	-57	-54	18	5.00	18.42	158
TMG_R	48	-60	-3	5.13	19.42	244
SPL/IPL_L	-15	-75	45	4.10	12.43	56
IPL_R	30	-54	45	4.93	17.91	28
Cerebelum_R	33	-3	-43	4.30	13.65	48
Cerebelum_L	-24	-48	-44	5.40	21.69	246
Insula/Putamen_R	30	15	-12	4.39	14.16	37

*Abbreviation*: *Bi* bilateral, *L* Left, *R* Right, *CAL* calcarine, *MOG* middle occipital cortex, *PoCG* postcentral gyrus, *PreCG* precentral gyrus, *LING* Ligual gyrus, *TMG* middle temporal gyrus, *IPL* inferior parietal lobule. * = a threshold of voxel-wise *p* < 0.001 uncorrected and cluster-level *p* < 0.05 after 3dFWHMx and 3dClustSim [AFNI (https://afni.nimh.nih.gov/) released in July 2017] was applied to correct for multiple comparisons

Compared with HCs, HDWI subjects exhibited significant decreased fALFF value in the bilateral CLA/TMG, right MOG/PoCG/IPL/LING and left PoCG/PreCG while increased fALFF in the bilateral CAL and right insular (voxel-wise *p* < 0.001 uncorrected and cluster-level *p* < 0.05 after 3dFWHMx and 3dClustSim) (see Fig. 2 and Table 3).

**Table 3:** The fALFF differences between groups the HDWI group and HCs (two sample *t* test)

Condition	Region	MNI coordinates X Y Z			Peak T value	Z value	Cluster size
HS < HC	CAL_Bi	3	-66	18	-4.72	-4.27	157
	MOG_R	33	-87	-3	-3.94	-3.66	28
	PoCG/PreCG_L	-42	-15	60	-5.05	-4.52	142
	PoCG_R	63	-9	36	-5.33	-4.72	112
	LING_R	12	-87	-6	-5.61	-4.92	113
	TMG_L	-60	-15	-9	-5.58	-4.90	62
	TMG_R	51	-66	18	-4.75	-4.29	56
	IPL_R	33	-51	42	-4.67	-4.23	43
HS > HC	Cerebelum_R*	33	-36	-35	3.69	3.45	13
	Cerebelum_L	-18	-51	-48	5.96	5.16	186
	Insula_R*	33	15	-11	4.60	4.18	15

*Abbreviation*: *Bi* bilateral, *L* Left, *R* Right, *CAL* calcarine, *MOG* middle occipital cortex, *PoCG* postcentral gyrus, *PreCG* precentral gyrus, *LING* Ligual gyrus, *TMG* middle temporal gyrus, *IPL* inferior parietal lobule. * = a threshold of voxel-wise *p* < 0.001 uncorrected and cluster-level *p* < 0.05 after 3dFWHMx and 3dClustSim [AFNI (https://afni.nimh.nih.gov/) released in July 2017] was applied to correct for multiple comparisons

Compared with HCs, HDWoI subjects exhibited significant decreased fALFF value in the bilateral CLA/MOG/PoCG/PreCG/TMG and left SPL while increased fALFF in the bilateral CAL and right insular ( voxel-wise *p* < 0.001 uncorrected and cluster-level *p* < 0.05 after 3dFWHMx and 3dClustSim) (see Fig. 2 and Supplementary Table 1).

However, when compared with HDWoI subjects, an opposite pattern of increased fALFF in the bilateral CAL/right MOG and decreased ALFF in the right cerebellum was observed in HDWI subjects ( voxel-wise *p* < 0.001 uncorrected and cluster-level *p* < 0.05 after 3dFWHMx and 3dClustSim) (see Fig. 2 and Table 4).

**Table 4:** The fALFF differences between the HDWI group and HDWoI group (two sample *t* test)

Condition	Region	MNI coordinates X Y Z			Peak T value	Z value	Cluster size
HS > HNS	CAL_Bi	0	-78	6	4.22	3.88	49
	MOG_R	33	-87	3	4.46	4.07	94
HS < HNS	Cerebelum_R*	36	-42	-48	-4.03	-3.73	15

*Abbreviation*: *Bi* bilateral, *R* Right, *CAL* calcarine, *MOG* middle occipital gyrus; * = a threshold of voxel-wise *p* < 0.001 uncorrected and cluster-level *p* < 0.05 after 3dFWHMx and 3dClustSim [AFNI (https://afni.nimh.nih.gov/) released in July 2017] was applied to correct for multiple comparisons

### Correlation analysis

**Fig. 3:**
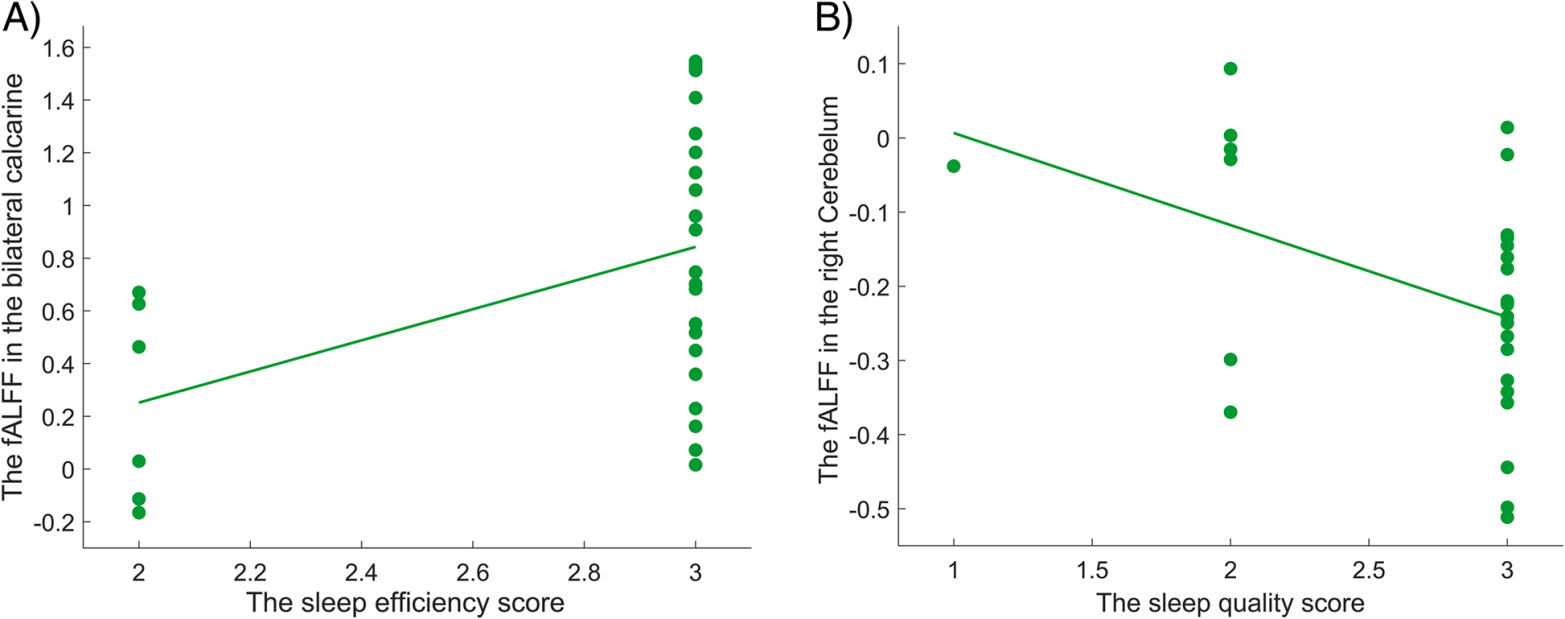
The correlation analyses between the ALFF features and clinical measurements in HDWI patients (**A**-**B**). A) Positive Correlation between the bilateral calcarine and the sleep efficiency subscore of PSQI( r = 0.460, *p* = 0.014); B) Negative correlation between the right cerebelum and the sleep quality subscore of PSQI( r = 0.421, *p* = 0.026)

Correlation analyses showed significant positive correlations of the fALFF values in the bilateral calcarine and the sleep efficiency subscore of PSQI ( *r* = 0.460, *p* = 0.014) in HDWI subjects. We also observed significant negative correlation between the right cerebelum and the sleep quality subscore of PSQI in HDWI subjects (*r* = 0.421, *p* = 0.026) (See Fig. 3).

### Machine learning results

**Fig. 4:**
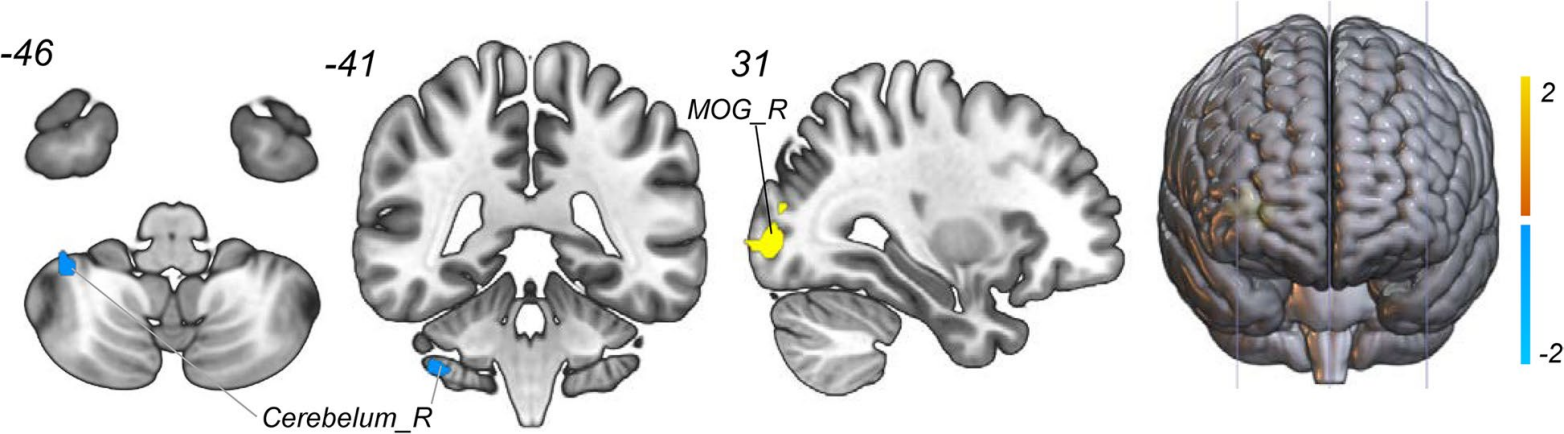
Discriminative brain regions. The discriminative regions included the right cerebelum and the right middle occipital gyrus. The color bar value represents the absolute value of the weight value of the brain regions. Yellow means positive weight and blue means negative weight

**Fig. 5:**
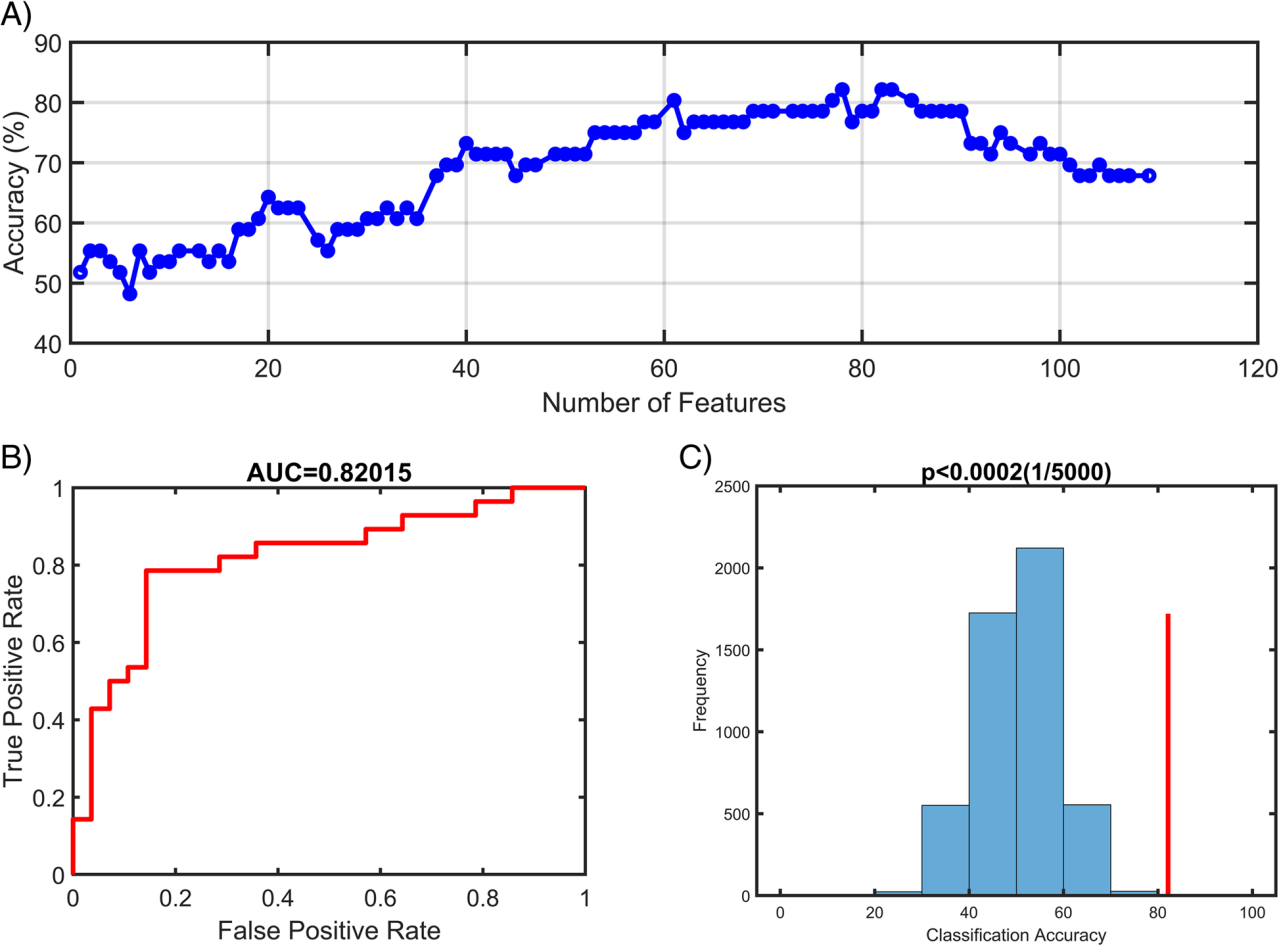
Classification performance of SVR model. **A**) The accuracy of classification with the increased number of features; when including 78 discriminative features, the highest accuracy of the classification model is 82.14%. **B**) Area under the curve of the classification model (AUC = 0.8202) with the highest accuracy. C) The result of the permutation test with the highest accuracy ( *p* < 0.0002)

As shown in Fig. 4 and Table 5, two clusters including the right MOG and right cerebellum were defined as meaningful classifying features that discriminate the HDWI subjects with noninsomniacs with high accuracy. Using the fALFF values voxels in these two clusters as input features to construct the SVR model and we found that the top 78 meaningful features significantly contributed to the classifier with the best discriminative ability (accuracy = 82.14%, sensitivity = 85.7%, specificity = 78.6% and AUC = 0.8202, respectively). The permutation analysis conducted 5,000 times showed that the classifier with 78 meaningful features was superior to the random classifiers ( *p* < 0.0002) (See Fig. 5).

**Table 5:** The weight of features to discriminate HDWI from HDWoI

Weight	Region	MNI coordinates X Y Z			Peak T value	Cluster size
Positive Weight	MOG_R	0	-78	6	1.06	63
Negative Weight	Cerebelum_R	37	-41	-46	-1.01	15

*Abbreviation*: *R* Right, *MOG* middle occipital gyrus

## Discussion

In the current study, we systematically investigated the fALFF pattern of HDWI and further identified reliable discriminative neural markers for classification by using a SVM model. Herein, three findings should be noted. First, when compared to HCs, patients in both groups (HDWI & HDWoI) exhibited reduced fALFF value in the bilateral CAL/MTG/PoCG, right IPL/MOG/LING, and left PreCG while increased ALFF in the bilateral cerebellum and right insular. However, an opposite cerebral activity pattern of increased fALFF in the bilateral CAL/right MOG and decreased fALFF in the right cerebellum was observed in HDWI subjects when compared with HDWoI subjects. Second, combined with selected fALFF features based on the inter-group comparisons, the SVM model achieved a good performance [accuracy = 82.14%, area under the curve (AUC) = 0.8202], and the consensus brain regions with the highest contributions to classification were located in the right MOG and right cerebellum. Third, correlation analyses showed significant positive correlation of the fALFF value in the bilateral calcarine with the sleep efficiency subscore of PSQI as well as the negative correlation between the right cerebellum and the sleep quality subscore of PSQI in HDWI subjects.

### Brain changes at the global level

In the study, we found lower fALFF in the CAL, MOG, PreCG, PoCG, LING, IPL and IPL in both HD groups (HDWI & HDWoI) compared with HCs. Our findings provide more data in support of previous evidence that the whole-brain structural and functional impairment among subjects undergoing hemodialysis [33–36]. Multiple factors secondary to renal dysfunction, including anemia, elevated C-reactive protein level, inflammation, electrolytes disturbances and accumulation of uremic toxins contribute to the damage of cerebral microvessels, and direct neuronal toxicity. In addition, conventional hemodialysis itself poses endothelial stress and injury on the already compromised vasculature system, consequently resulting in the declines of the brain function in HD subjects [37–39]. A large body of neuroimaging research in patients on HD [16, 40, 41] have discovered the reduced neural activity, changed cerebral perfusion in widespread brain regions, and related psychological impairment in HD patients. Particularly, a most recent multimodal fMRI study employed fMRI technique combined with arterial spin labeling (ASL) technique—a MRI method for evaluating the cerebral blood flow- to investigate the neurovascular coupling (NVC) mechanism of patients undergoing hemodialysis, and found significantly decreased ALFF-CBF values in several brain regions compared with the HCs [41]. Therefore, given the previous evidence of the abnormal brain activities and structural impairment in widely distributed regions, together with our findings of decreased fALFF in multiple regions, we believed that our study further confirmed the fact that the hemodialysis may have a negative effect on the global cerebral function on HD population including HDWI group to some extent.

### MOG/CAL involvement

We also observed greater fALFF values in HDWI subjects in the right MOG and bilateral CAL compared with HDWoI subjects, despite a significant decreased fALFF in such regions detected when compared with HCs. The increased neuronal activity, in other words, reduced deactivation in MOG/CAL was theorized to reflect an hyperactivited state of these regions in HDWI population in the context of the overall declines of brain function in HD subjects. These findings support a multicausal theoretical account of sensory over-activated hyperarousal of insomnia, which believes that a hypersensitivity to external stimuli during sleep might be driven by an overactivity of the somatic and cognitive cortical regions as visual network (MOG/CAL), sensory motor network (SMN) or other sensory -related regions. Mounting evidence has revealed distinct visual network activation including MOG and CAL in response to sleep-related visual processing in the chronic insomnia subjects and in cases of sleep deprivation [42–44]. For example, Zhou FQ, et, al. [45] used ALFF method to examine the local intrinsic activity in chronic primary patients and observed increased ALFF values for neuronal activity in the second visual cortex (MOG) and CAL, suggesting their involvements in insomnia. Similarly, another rsfMRI study [14] reported that chronic insomnia patients with difficulty in initiating or maintaining sleep showed disrupted brain network topology of SMN with visual networks. Moreover, the MOG has been reported to participate in spatial processing of auditory and tactile stimuli, and category-selective, attention-modulated unconscious processing, which might link the daytime impairments reported in HDWI patients, like spatial memory decline and distraction [46]. Thus, the modification of MOG might also reflect the psychological conditions following insomnia such as memory or cognitive deficits. Overall, given the various functions of the CAL/MOG, it is difficult to distinguish whether it is responsible for sleep itself or sleep-loss conditions, but it is clear that the CAL/MOG indeed play a vital role in the HDWI modulation system. Importantly, this hypothesis yielded a consistent association with our other findings, that features in the right MOG ranked highly in the SVM classifier, and the increased fALFF in the bilateral CAL was also positively associated with clinical measurements (sleep efficiency subscore of PSQI).

### Cerebellum involvement

Another highly ranked feature in the classification model was the decreased fALFF in the Cerebellum, which is an important finding in light of accumulative evidence for cerebellar involvement in its non-motor function including cognitive, emotion and sleep over the past decades [47–49]. This is actually surprising given that, until quite recently, this brain structure was thought to contribute primarily to the planning and execution of movements. The cerebellum is proposed to interconnect an extensive network with cortical and subcortical areas to form a feedback loop in facilitating a series of motor-related behaviors [50, 51]. Evidence from fMRI [52, 53] showed anatomical projections from the cerebellum to the thalamic, limbic, striatal, and cerebrocortical regions. Such anatomical connections may provide an important substrates for cerebellar involvement in cognition and emotion. Functionally, Early in 1988, Petersen and colleagues [54] conducted a pioneering PET study to measure brain function while people viewed words and engaged in progressively more elaborate task. They initially concluded that “The different response locale from cerebellar motor activation and the presence of the activation to the generate use subtractions argue for a ‘cognitive’, rather than a sensory or motor computation being related to this activation”. Since then a mountain of compelling evidence has been generated that the human cerebellar responds to multiple domains of cognitive tasks and emotions [55, 56]. Particularly, many meta-analytic results [57, 58] provide further comprehensive insights into cerebellar involvement and elucidate its role in higher cognition. Most importantly, a recent fMRI study detected abnormal spontaneous regional brain activity in the bilateral cerebellum posterior lobes in primary insomnia, suggesting its involvement in insomnia disorders [14]. Taken together, these results not only provide sufficient evidence to solidify the concept of the cerebellum’s contribution to non-motor functions, but also imply that the altered activity in cerebellum underlie the impairment in insomnia involving cognitive and emotional dysfunction.

As a special group, individuals on hemodialysis were more dissatisfied with their physical and mental conditions compared to the healthy controls, resulting in negative mood, anxiety, and depression [5, 59]. Actually, such conditions also can be seen in our study with higher SAS/SDS scores and lower SF-36 scores compared with the non-insomniacs (*p* < 0.05). These negative emotional factors may contribute to the development of insomnia as independent factors, while sleep disturbance would in turn lead to daytime sleepiness, fatigue, social isolation, increasing depressive/anxious disorders and cognitive impairment [60, 61]. Also, as mentioned above, other end-stage renal disease (ESRD)—and HD- related factors are demonstrated to be closely associated with compromised cognitive function as well. The increased fALFF value detected in HDWI patients in our study thus suggested that the disturbed nocturnal sleep in patients on hemodialysis may have a harmful effect on the cerebellum. What’s more, we also observed negative correlation of altered fALFF values with the sleep quality subscore of PSQI, and that altered fALFF has a high classification rank in SVR model, reflecting the discriminative capacity of cerebellum for HDWI. However, it is worth noting that although the crucial role of cerebellum involved in the emotional and cognitive processing has been confirmed, its involvement in HDWI is still novel. This is the first we noted such brain region, and our team provides a initial exploration on highlighting its influence on HDWI and—possibly—emotional and cognitive conditions.

### SVM classifier performance

This is the first we constructed a SVM classifier to find a promising model for HDWI classification. Although previous studies have offered insights into brain functional and structural abnormalities of neurological diseases including primary insomnia using traditional group-level fMRI analysis, they could not translated into diagnostic or predictive neural markers for such neurological diseases, especially in HD cohort. Our study just filled the gap in such cohort by constructing a highly discriminative SVM classifier, which was efficient to provide preliminary support to develop the individualized therapeutic aid for HDWI. In fact, The ability to advise clinicians and patients accurately regarding the chances of proper therapy is of great importance, particularly as improper therapy is an occupation of medical resource waste and may has some side reactions. Our findings not only confirmed that functional neuroimaging data has the potential to serve as discriminative markers for HDWI, but also provided further evidence that rsfMRI in conjunction with SVM model could help support a classification of diseases, which may be important to elucidate the neural mechanisms of HDWI and pave the way towards more personalized interventions.

There are still some limitations to be noted in this study. Firstly, the sample size was relatively small ( HDWI = 28, HDWoI = 28), and a larger sample size will be supplemented to increase data reliability. Secondly, all the subjects came from the same center, and we will continue to perform a multicenter study to validate our results. Third, the rs-fMRI datasets were collected from the subjects in the relatively old age group (average 59.86, 55.11, 51.96 age, respectively). To generalize analysis results, other datasets collected from more younger or wide range of age subjects were needed. Thus, in the future, we will carry on further investigations to address these issues.

## Conclusions

To conclude, besides describing the global aberrant intrinsic activity pattern of individuals on HD, our study for the first time revealed that the HDWI patients exhibited abnormal fALFF in the right MOG, bilateral CAL and the right cerebellum compared to HDWoI. Moreover, the fALFF features in the right MOG and right cerebellum contributed most to the group discrimination in the machine learning classifier, suggesting that these features could be identified as reliable markers in discriminating the insomniacs on hemodialysis from those non-insomniacs.

## Supplementary Information

**Additional file 1.**

## Data Availability

The data and materials underlying this article will be shared on reasonable request to the corresponding author.

## References

[1] Kir S, Kirhan İ, Dilek M (2021). Prevalence of Sleep Disorders and Related Factors in Individuals Undergoing Hemodialysis. Cogn Behav Neurol.

[2] Unruh M, Cukor D, Rue T (2020). Sleep-HD trial: short and long-term effectiveness of existing insomnia therapies for patients undergoing hemodialysis. BMC Nephrol.

[3] Knezevic MZ, Djordjevic VV, Jankovic SM, Vidojko MD (2013). Influence of dialysis modality and membrane flux on insomnia severity in haemodialysis patients. Nephrology (Carlton).

[4] Elder SJ, Pisoni RL, Akizawa T (2008). Sleep quality predicts quality of life and mortality risk in haemodialysis patients: results from the Dialysis Outcomes and Practice Patterns Study (DOPPS). Nephrol Dial Transplant.

[5] Wang R, Tang C, Chen X (2016). Poor sleep and reduced quality of life were associated with symptom distress in patients receiving maintenance hemodialysis. Health Qual Life Outcomes.

[6] Hamzi MA, Hassani K, Asseraji M, El Kabbaj D (2017). Insomnia in hemodialysis patients: a multicenter study from morocco. Saudi J Kidney Dis Transpl.

[7] Uzun S, Kozumplik O, Jakovljević M (2010). Biserka Sedić. Side effects of treatment with Benzodiazepines. Psychiatr Danub..

[8] Yeh CY, Chen CK, Hsu HJ (2014). Prescription of psychotropic drugs in patients with chronic renal failure on hemodialysis. Ren Fail.

[9] Kim SJ, Lee YJ, Kim N (2017). Exploration of changes in the brain response to sleep-related pictures after cognitive behavioral therapy for psychophysiological insomnia. SciRep.

[10] Zhao H, Li D, Yang Y, Liu YT, Li J, Mao J (2019). Auricular plaster therapy for comorbid insomnia: a systematic review and meta-analysis of randomized controlled trials”. Evid Based Complement Alternat Med.

[11] Ashina S, Bentivegna E, Martelletti P, Eikermann-Haerter K (2011). Structural and functional brain changes in migraine. Pain Ther.

[12] Kim N, Won E, Cho SE, Kang CK, Kang SG (2021). Thalamocortical functional connectivity in patients with insomnia using resting-state fMRI. J Psychiatry Neurosci.

[13] Huang G, Fang Y, Zhang W (2022). Altered thalamic functional connectivity and cerebral blood flow in insomnia disorder: a resting-state functional magnetic resonance imaging study. Clin Imaging.

[14] Li C, Ma XF, Dong MS (2016). Abnormal spontaneous regional brain activity in primary insomnia: a resting-state functional magnetic resonance imaging study. Neuropsychiatr Dis Treat.

[15] Su H, Fu S, Liu M (2022). Altered Spontaneous Brain Activity and Functional Integration in Hemodialysis Patients With End-Stage Renal Disease. Front Neurol.

[16] Zhang D, Chen Y, Wu H (2021). Associations of the Disrupted Functional Brain Network and Cognitive Function in End-Stage Renal Disease Patients on Maintenance Hemodialysis: A Graph Theory-Based Study of Resting-State Functional Magnetic Resonance Imaging. Front Hum Neurosci.

[17] Zheng JH, Sun Q, Wu XX (2022). Brain Micro-Structural and Functional Alterations for Cognitive Function Prediction in the End-Stage Renal Disease Patients Undergoing Maintenance Hemodialysis. Front Neurosci.

[18] Xu M, Calhoun V, Jiang R, Yan W, Sui J (2021). Brain imaging-based machine learning in autism spectrum disorder: methods and applications. J Neurosci Methods.

[19] Zheng J, Wei X, Wang J, Lin H, Pan H, Shi Y (2021). Diagnosis of Schizophrenia Based on Deep Learning Using fMRI. Comput Math Methods Med.

[20] Ueno T, Ichikawa D, Shimizu Y (2022). Comorbid insomnia among breast cancer survivors and its prediction using machine learning: a nationwide study in Japan. Jpn J Clin Oncol.

[21] Noble WS (2006). What is a support vector machine?. Nat biotechnol.

[22] Jan Z, Ai-Ansari N, Mousa O (2021). The Role of Machine Learning in Diagnosing Bipolar Disorder: Scoping Review. J Med Internet Res.

[23] Shi DF, Zhang HR, Wang GS (2022). Machine Learning for Detecting Parkinson's Disease by Resting-State Functional Magnetic Resonance Imaging: A Multicenter Radiomics Analysis. Front Aging Neurosci.

[24] Zhang B, Jung M, Tu Y (2019). Identifying brain regions associated with the neuropathology of chronic low back pain: a resting-state amplitude of low-frequency fluctuation study. Br J Anaesth.

[25] He D, Ren D, Guo Z, Jiang B (2022). Insomnia disorder diagnosed by resting-state fMRI-based SVM classifier. Sleep Med.

[26] American Psychiatric Association (2013). Diagnostic and Statistical Manual of Mental Disorders (DSM-5 ® ).

[27] Buysse DJ, Reynolds CF, Monk TH, Berman SR, Kupfe DJ (1989). The Pittsburgh Sleep Quality Index: a new instrument for psychiatric practice and research. Psychiatry Res.

[28] Hao W, Tang Q, Huang X (2021). Analysis of the prevalence and influencing factors of depression and anxiety among maintenance dialysis patients during the COVID-19 pandemic. Int Urol Nephrol.

[29] Mingardi G, Cornalba L, Cortinovis E, Ruggiata R, Mosconi P, Apolone G (1999). Health-related quality of life in dialysis patients. A report from an Italian study using the SF-36 Health Survey. DIA-QOL Group. Nephrol Dial Transplant..

[30] Yan CG, Wang XD, Zuo XN, Zang YF (2016). DPABI: Data Processing and Analysis for (Resting-State) Brain Imaging. Neuroinformatics.

[31] Feng M, Zhang Y, Wen Z (2022). Early Fractional Amplitude of Low Frequency Fluctuation Can Predict the Efficacy of Transcutaneous Auricular Vagus Nerve Stimulation Treatment for Migraine Without Aura. Front Mol Neurosci.

[32] Chang C, Lin C (2011). LIBSVM: a library for support vector machines. ACM Trans Intell Syst Technol.

[33] Karakizlis H, Bohl K, Ziemek J, Dodel R, Hoyer J (2022). Assessment of cognitive impairment and related risk factors in hemodialysis patients. J Nephrol.

[34] Chen HJ, Qi R, Kong X (2015). The impact of hemodialysis on cognitive dysfunction inpatients with end-stage renal disease: a resting-state functional MRI study. Metab Brain Dis.

[35] Wang H, Huang L, Wu G (2022). Regional cerebral gray matter atrophy is associated with cognitive impairment in hemodialysis patients: a cross-sectional and longitudinal voxel-based morphological MRI study. Brain Imaging Behav.

[36] Zhang R, Liu K, Yang L (2015). Reduced white matter integrity and cognitive deficits in maintenance hemodialysis ESRD patients: a diffusion-tensor study. Eur Radiol.

[37] Chen HY, Chiu YL, Hsu SP (2010). Elevated C-reactive protein level in hemodialysis patients with moderate/severe uremic pruritus: a potential mediator of high overall mortality. QJM.

[38] Taraz M, Khatami MR, Hajiseyedjavadi M (2013). Association between antiinflammatory cytokine, IL-10, and sleep quality in patients on maintenance hemodialysis. Hemodial Int.

[39] Hernandez L, Ward LJ, Arefin S, et al. GOING-FWD Collaborators, et al. Blood-brain barrier and gut barrier dysfunction in chronic kidney disease with a focus on circulating biomarkers and tight junction proteins.Sci Rep. 2022;12(1):4414. 10.1038/s41598-022-08387-7 PMC8924178 35292710

[40] Lu H, Gu Z, Xing W (2019). Alterations of Default mode functional connectivity in individuals with end-stage renal disease and mild cognitive impairment. BMC Nephrol.

[41] Li P, Mu J, Ma X (2021). Neurovascular coupling dysfunction in end-stage renal disease patients related to cognitive impairment. J Cereb Blood Flow Metab.

[42] Riemann D, Spiegelhalder K, Feige B (2010). The hyperarousal model of insomnia: A review of the concept and its evidence. Sleep Med Rev.

[43] Mendoza JF, Li Y, Vgontzas AN (2016). Insomnia is associated with cortical hyperarousal as early as adolescence. Sleep.

[44] Nofzinger EA, Buysse DJ, Germain A, Price JC, Miewald JM, Kupfer DJ (2004). Functional neuroimaging evidence for hyperarousal in insomnia. Am J Psychiatry.

[45] Zhou FQ, Huang SH, Zhuang Y, Gao L, Gong HH (2016). Frequency-dependent changes in local intrinsic oscillations in chronic primary insomnia: A study of the amplitude of low-frequency fluctuations in the resting state. Neuroimage Clin.

[46] Drummond SP, Walker M, Almklov E, Campos M, Anderson DE, Straus LD (2013). Neural correlates of working memory performance in primary insomnia. Sleep.

[47] Sathyanesan A, Zhou J, Scafidi J, Heck DH, Sillitoe RV (2019). Emerging connections between cerebellar development, behavior, and complex brain disorders. Nat Rev Neurosci.

[48] Adamaszek M, Agata FD, Ferrucci R (2017). Consensus paper: cerebellum and emotion. Cerebellum.

[49] Randy LB (2013). The cerebellum and cognitive function: 25 years of insight from anatomy and neuroimaging. Neuron.

[50] Mackie S, Philip S, Rhoshel L (2007). Cerebellar development and clinical outcome in attention deficit hyperactivity disorder. Am J Psychiatry.

[51] Leggio M, Olivito G (2018). Topography of the cerebellum in relation to social brain regions and emotions. Handbook of Clinical Neurology, Elsevier B.V..

[52] Middleton FA, Strick PL (2001). Cerebellar projections to the prefrontal cortex of the primate. J Neurosci.

[53] Guell X, Gabrieli JDE, Schmahmann JD (2018). Triple representation of language, working memory, social and emotion processing in the cerebellum: convergent evidence from task and seed-based resting-state fMRI analyses in a single large cohort. Neuroimage.

[54] Petersen SE, Fox PT, Posner MI, Mintun M, Raichle ME (1989). Positron emission tomographic studies of the processing of singe words. J Cogn Neurosci.

[55] Allen G, McColl R, Barnard H, Ringe WK, Fleckenstein J, Cullum CM (2005). Magnetic resonance imaging of cerebellar-prefrontal and cerebellar-parietal functional connectivity. Neuroimage.

[56] King M, Hernandez-Castillo CR, Poldrack RA, Ivry RB, Diedrichsen J (2019). Functional boundaries in the human cerebellum revealed by a multi-domain task battery. Nat Neurosci.

[57] Stoodley CJ, Schmahmann JD (2009). Functional topography in the human cerebellum: a meta-analysis of neuroimaging studies. Neuroimage.

[58] Keren-Happuch E, Chen SHA, Ho MHR, Desmond JE (2014). A meta-analysis of cerebellar contributions to higher cognition from PET and fMRI studies. Hum Brain Mapp.

[59] Scherer JS, Combs SA, Brennan F (2017). Sleep disorders, restless legs syndrome, and uremic pruritus: diagnosis and treatment of common symptoms in dialysis patients. Am J Kidney Dis.

[60] Theofilou P (2013). Association of insomnia symptoms with kidney disease quality of life reported by patients on maintenance dialysis. Psychol Health Med.

[61] Rai M, Rustagi T, Rustagi S, Kohli R (2011). Depression, insomnia and sleep apnea in patients on maintenance hemodialysis. Indian J Nephrol.

