# Supplementary material for: Identification of discriminative neuroimaging markers for patients on hemodialysis with insomnia: a fractional amplitude of low frequency fluctuation-based machine learning analysis
Source: BMC Psychiatry. 2023 Jan 4;23:9. doi: 10.1186/s12888-022-04490-1 (PMC9811801; doi:10.1186/s12888-022-04490-1)
Supplement: Supplementary file 1 — Additional file 1. [file 12888_2022_4490_MOESM1_ESM.doc]

| Condition | Region | MNI coordinates  X Y Z | | | Peak  T value | Z  value | Cluster  size |
| --- | --- | --- | --- | --- | --- | --- | --- |
| HNS< HC | CAL_Bi | 3 | -69 | 18 | -7.42 | -6.06 | 561 |
|  | MOG_L | -42 | -66 | 3 | -6.31 | -5.39 | 380 |
|  | MOG_R | 33 | -75 | 27 | -6.25 | -5.34 | 227 |
|  | PoCG/PreCG_L  PoCG/PreCG_R | -51  60 | -18  -9 | 60  36 | -5.99  -7.54 | -5.17  -6.13 | 227  265 |
|  | TMG_L  TMG_R  SPL_L | -63  63  -24 | -21  -42  -63 | 8  3  48 | -5.06  -6.26  -5.04 | -4.53  -5.36  -4.51 | 56  227  68 |
| HNS > HC | Cerebelum_R | 33 | -38 | -43 | 5.06 | 4.53 | 51 |
|  | Cerebelum_L | -27 | -48 | -45 | 5.87 | 5.09 | 236 |
|  | Insula_R/Putamen_R | 30 | 15 | -12 | 5.06 | 4.53 | 30 |

**Supplementary Table 1.** Brain regions showing differences in fALFF between the HDWoI and HC group

Abbreviation: Bi = bilateral; L: Left; R = Right; CAL= calcarine; MOG = middle occipital cortex; PoCG = postcentral gyrus; PreCG = precentral gyrus; LING = Ligual gyrus; TMG = middle temporal gyrus; SPL = superior parietal lobule.
